# Diagnostic utility of coronary artery calcium score percentiles and categories to exclude abnormal scans and relevant ischemia in rubidium positron emission tomography

**DOI:** 10.3389/fcvm.2024.1467916

**Published:** 2024-09-23

**Authors:** Simon M. Frey, Gabrielle Huré, Jan-Philipp Leibfarth, Kathrin Thommen, Melissa Amrein, Klara Rumora, Ibrahim Schäfer, Federico Caobelli, Damian Wild, Philip Haaf, Christian E. Mueller, Michael J. Zellweger

**Affiliations:** ^1^ Department of Cardiology, University Hospital Basel, University of Basel, Basel, Switzerland; ^2^ Cardiovascular Research Institute Basel (CRIB), University Hospital Basel, University of Basel, Basel, Switzerland; ^3^ Department of Nuclear Medicine, University Hospital Bern, University of Bern, Bern, Switzerland; ^4^ Division of Nuclear Medicine, University Hospital Basel, University of Basel, Basel, Switzerland

**Keywords:** coronary artery disease (CAD), coronary artery calcium score (CACS), patient stratification, ischemia, positron emission tomography (PET), gatekeeper, percentile

## Abstract

**Background:**

Despite clinical suspicion, most non-invasive ischemia tests for coronary artery disease (CAD) reveal unremarkable results. Patients with a coronary artery calcium score (CACS) of zero rarely have an abnormal positron emission tomography (PET) and could be deferred from further testing. However, most patients have some extent of coronary calcification.

**Objectives:**

CACS percentiles could be useful to exclude abnormal perfusion in patients with CACS >0, but data from patients with ^82^Rb PET are lacking. The aim of this study was to assess the diagnostic utility of CACS percentiles in comparison to zero calcium and absolute CACS classes.

**Methods:**

Consecutive patients with suspected CAD (*n* = 1,792) referred for ^82^Rb PET were included and analyzed for abnormal PET (SSS ≥4) and relevant ischemia (>10% myocardium). Test characteristics were calculated.

**Results:**

The mean age was 65 ± 11 years, 43% were female, and typical angina was reported in 21%. Abnormal PET/relevant ischemia (>10%) were observed in 19.8%/9.3%. Overall, the sensitivity/negative predictive value (NPV) of a <25th percentile CACS to rule out abnormal PET and relevant ischemia were 93.0%/95.7% and 98.2%/99.5%, respectively. The sensitivity/NPV of CACS 1–9 to rule out abnormal PET and relevant ischemia were 96.0%/91.8% and 97.6%/97.6%, respectively. Except for patients <50 years old, sensitivity for abnormal PET was >90.9% in all age groups.

**Conclusion:**

In patients >50 years, the <25th percentile and CACS 1–9 had good test characteristics to rule out abnormal PET and relevant ischemia (>10%). They could be used to extend the scope of application of CACS 0 by 8%–10% to 32%–34% overall of patients who could be deferred from further testing.

## Graphical Abstract

Use of coronary artery calcium score (CACS) <25th percentile and CACS 1–9 for detection of abnormal positron emission tomography (PET) and relevant ischemia (>10%). The study included patients with suspected CAD referred for ^82^Rb-PET with an available CACS. Test characteristics of <25th percentile, CACS 1–9 and CACS 0 are displayed in the table. Panels **(A)** and **(B)** show the proportion of patients with <25th percentile (and CACS >0) and CACS 1–9, respectively, which could additionally be triaged using CACS in addition to CACS 0. The bar charts in panels **(C)** and **(D)** indicate the prevalence of abnormal PET (SSS ≥4) and relevant ischemia (>10%) according to CACS percentile and CACS class. With incremental percentile and CACS class, the prevalence of abnormal PET/relevant ischemia (>10%) increased significantly (*p* < 0.001 each).

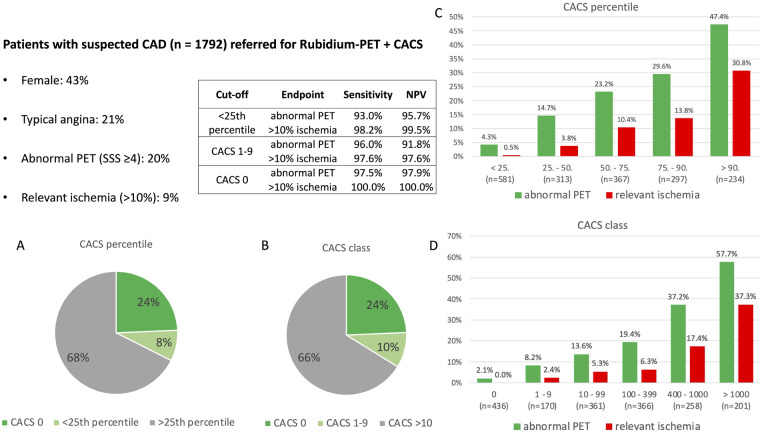

## Introduction

Coronary artery disease (CAD) is very common and responsible for significant morbidity, mortality, and healthcare costs (1). Different non-invasive tests are available and can be used for diagnosis and risk stratification. Current European Society of Cardiology guidelines recommend functional testing for the detection of prognostically relevant myocardial ischemia in patients with moderate-high pre-test probability (PTP) (2). Because the prevalence of CAD in patients referred for cardiac imaging has declined over the past few decades, the proportion of low-risk test results has significantly increased (3). Hence, unremarkable non-invasive ischemia tests are often reported to be as high as 60%–79%, resulting in unnecessary exposure of patients to medication, contrast and stress agents, radiation, and high healthcare costs (4–7). Thus, there is a need for optimized patient preselection.

Coronary artery calcium score (CACS) is a simple, widely available, and inexpensive test to visualize and quantify the amount of coronary artery calcification (CAC) (8). Due to its excellent sensitivity and negative predictive value (NPV), it is a good gatekeeper candidate prior to advanced testing (9). In particular, the absence of CAC [or “zero calcium score” (ZCS)] can be used to rule out obstructive CAD or myocardial ischemia with satisfactory certainty (7, 10–12) and is associated with an excellent prognosis (12–15).

However, most patients with suspected CAD have some extent of CAC and, consequently, ZCS cannot be used in the majority of cases [prevalence of ZCS: 24% in this study cohort, 35% in PROMISE (15), and 36% in SCOT-HEART (16)].

There are several studies that have documented the correlation of myocardial ischemia with increasing absolute CACS values, but only one study examined CACS quartiles (17–20).

In an older and smaller single photon emission tomography (SPECT) study, the prevalence of abnormal scan results or moderate-to-severe ischemia was reported to be very low in the first and second CACS quartile (<0.5% and <2%, respectively) (20). However, similar studies on CACS quartiles (or percentiles) as a gatekeeper to exclude myocardial ischemia in positron emission tomography (PET) are lacking.

Hence, the objectives of this study were the following: (1) assess the diagnostic utility of CACS percentiles (in particular, the <25th percentile) to rule out abnormal scan results and prognostically significant ischemia in ^82^Rb PET, (2) compare the diagnostic properties with traditionally used absolute CACS classes, and (3) analyze the findings in different age groups.

## Methods

### Study design and patient selection

**Figure 1:**
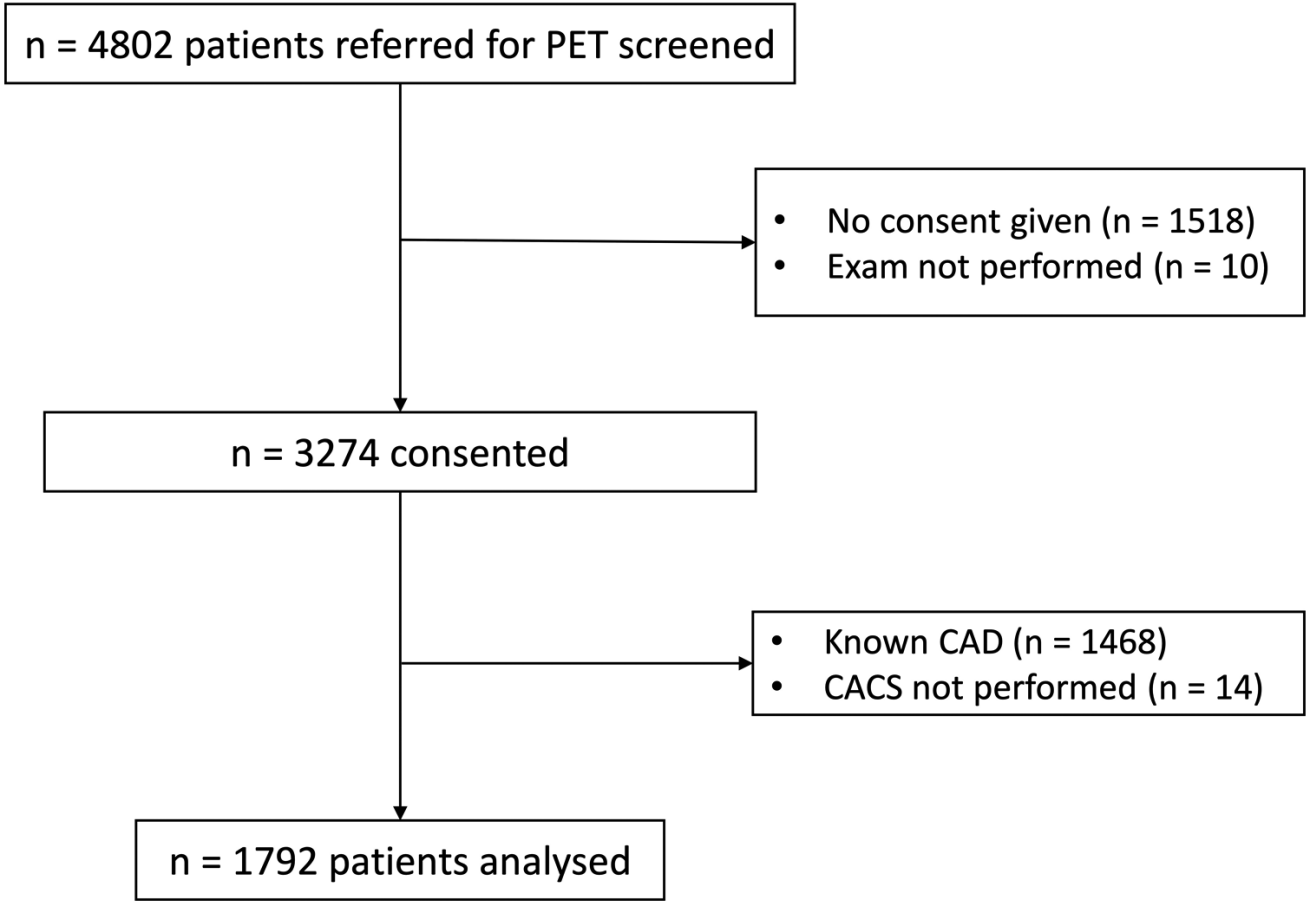
Flow chart of the patient selection. All patients referred for PET were screened. If they consented, they were included in the cohort. For this project, patients with known CAD or no available CACS were excluded.

Consecutive patients referred for an ^82^Rb-PET scan at our tertiary center (University Hospital Basel) between July 2018 and October 2022 were screened and invited to participate in this prospective cohort study. If patients consented to the use of their healthcare data, they were included. For this project, patients with known CAD were excluded. The study flow is illustrated in Figure 1.

The study was carried out according to the principles of the Declaration of Helsinki and was approved by the local ethics committee (Ethikkommission der Nordwest- und Zentralschweiz EKNZ, project ID: PB_2018-00076/EK 67/08).

### Imaging protocol and analysis

Imaging protocols were used as described previously (5, 10). In short, patients were instructed to withhold caffeine-containing products for 24 h before the test. A 3D-PET/CT scanner was used (Biograph mCT, Siemens Healthineers, Erlangen, Germany). A low-dose CT scan was obtained for attenuation correction [increment 0.6 mm, soft-tissue reconstruction kernel, 120 keV, CAREDOSE 4D (Siemens Healthineers, Erlangen, Germany)]. Subsequently, a second, ECG-triggered non-enhanced low-dose CT during breath hold was acquired for CACS (120 kV, 40 mAs, rotation time 2.1/s, Matrix 128 × 128, slice thickness 3 mm). Patient-specific dose modulation was automatically adapted by the scanner software according to the localizer image (CAREDOSE).

Thereafter, ^82^Rb-chloride was intravenously injected in a weight-adjusted manner (30–40 mCi) both at rest and during stress. Rest was always performed first. Stress was pharmacologically induced with adenosine (140 µg/kg/min for 6 min). If contraindications (mostly allergic asthma) or personal preferences were present, regadenoson was used instead (400 µg single dose). Patients were monitored according to current guidelines (21).

Dynamic, ECG-gated PET images were acquired both at rest and during stress over 7 min in list mode starting with tracer injection and then reconstructed as described in the Supplementary Material. Reconstructed images were displayed and visually inspected with QGS-QPS software included in the SyngoVia package (Siemens Healthineers, Erlangen, Germany). CACS was calculated with the Coronary CT tool included in the SyngoVia package according to the Agatston method, using 130 HU as the threshold as previously published (8).

The images were analyzed and interpreted by an experienced board-certified nuclear medicine physician and cardiologist as a joint read, reaching a consensus. A visual semi-quantitative 17-segment model with a 5-point scale (0: normal tracer uptake, 4: no tracer uptake) was used to calculate the summed stress (SSS), rest (SRS), and difference scores (SDS = SSS − SRS). An SSS ≥4 was considered an abnormal PET. An SDS ≥7 was considered as the threshold for a relevant ischemia, consistent with ≥10% of the left ventricular myocardium being involved, as suggested in the guidelines on the criteria to consider an invasive evaluation with subsequent revascularization (2). An SDS ≥2 was considered as small ischemia. Age- and sex-specific CACS percentiles according to Hoff et al. (22) (<25th, 25–50th, 50–75th, 75–90th, and >90th percentiles) and absolute CACS categories (0, 1–9, 10–99, 10–399, 400–999, >1,000) were used.

Myocardial blood flow was automatically calculated with SyngoVia (Siemens Healthineers, Erlangen, Germany) and approved by the readers. A global myocardial flow reserve <2.0 was considered microvascular dysfunction.

### Statistical analysis

Normally distributed continuous variables are reported as mean ± standard deviation (SD) and statistical testing was performed with an unpaired *t*-test or ANOVA. Non-normally distributed continuous variables are reported as median with interquartile range (IQR) and statistical testing was performed with the Wilcoxon test. Categorical variables are displayed using frequencies and percentages and were compared using the Chi-squared test or Fisher's exact test where appropriate. A *p*-value <0.05 was considered statistically significant.

Endpoints were defined as an SSS ≥4 and SDS ≥7. Sensitivity, specificity, positive and negative predictive value (PPV and NPV), positive and negative likelihood ratio (PLR and NLR), diagnostic odds ratio (DOR), and false negative and false positive rate (FNR, FPR) were calculated. The analysis was also repeated for different age groups and sex. Receiver operating characteristic (ROC) curve analysis was performed to determine the area under the curve (AUC). Comparison between the AUCs of CACS percentile, CACS category, and continuous CACS was performed using the DeLong method. For this calculation, a Bonferroni corrected *p*-value of <0.0167 (*α* = 0.05/3, given three comparisons) was considered significant.

A binary logistic regression analysis was performed by inserting clinically relevant variables into the model [age, sex, symptoms, body mass index (BMI), cardiovascular risk factors, left bundle branch block (LBBB), Q wave, repolarization disturbance, CACS percentile].

An SSS ≥4 is a composite endpoint of scarring and ischemia which is widely used in the literature. For safety analysis, the analyses described above were repeated using small ischemia (SDS ≥2) as the endpoint. Statistical analyses were performed using SPSS™ (version 28.0.1.0) and RStudio (using R version 4.2.2).

## Results

### Patient population

A total of 1,792 patients were analyzed for this study. The mean age was 65 ± 11 years and 43% were female. Typical and atypical angina were reported in 21% and 25%, respectively. Female patients were more often symptomatic (78.9% vs. 68.0%, *p* < 0.001). Diabetes, smoking history, and hypercholesteremia were more frequent in male patients. Median CACS was 74 (1–413) and was significantly higher in males than in females [148 (11–621) vs. 16 (0–181), *p* < 0.001]. Detailed baseline characteristics are shown in Table 1. Abnormal PET and relevant ischemia (>10%) were present in 19.8% (*n* = 355) and 9.3% (*n* = 166), respectively. Microvascular dysfunction was observed in 16.0% (*n* = 283) of the patients.

**Table 1:** Baseline characteristics.

	Overall (*n* = 1,792)	Male (*n* = 1,030)	Female (*n* = 762)	*p*-value
Age (years)	65.4 (11.0)	64.6 (10.8)	66.6 (11.1)	<0.001
BMI (kg/m^2^)	28.1 (5.8)	28.2 (5.1)	27.9 (6.5)	0.287
Stroke (%)	76 (4.2)	45 (4.4)	31 (4.1)	0.846
COPD (%)	82 (4.6)	51 (5.0)	31 (4.1)	0.441
Peripheral artery disease (%)	63 (3.5)	39 (3.8)	24 (3.1)	0.553
Dialysis (%)	16 (0.9)	13 (1.3)	3 (0.4)	0.093
Cancer (%)	191 (10.7)	112 (10.9)	79 (10.4)	0.790
Risk factors				
Hypertension (%)	788 (44.0)	469 (45.5)	319 (41.9)	0.134
Hypercholesterolemia (%)	645 (36.0)	391 (38.0)	254 (33.3)	0.049
Diabetes (%)	375 (20.9)	246 (23.9)	129 (16.9)	<0.001
Smoking history (%)	1,025 (57.2)	659 (64.0)	366 (48.0)	<0.001
Family history (%)	206 (11.5)	112 (10.9)	94 (12.3)	0.376
Symptoms (%)				
Symptomatic	491 (27.4)	330 (32.0)	161 (21.1)	<0.001
Non-anginal	204 (11.4)	101 (9.8)	103 (13.5)	
Atypical angina	445 (24.8)	228 (22.1)	217 (28.5)	
Typical angina	371 (20.7)	190 (18.4)	181 (23.8)	
Dyspnea	281 (15.7)	181 (17.6)	100 (13.1)	
ECG findings				
Sinus rhythm (%)	1,644 (91.7)	924 (89.7)	720 (94.5)	<0.001
LBBB (%)	78 (4.4)	42 (4.1)	36 (4.7)	0.585
Q wave (%)	70 (3.9)	47 (4.6)	23 (3.0)	0.217
Abnormal repolarization (%)	222 (12.4)	125 (12.1)	97 (12.7)	0.808

BMI, body mass index; COPD, chronic obstructive pulmonary disease; LBBB, left bundle branch block.

Baseline characteristics of included patients are stratified by sex. Values are displayed as mean (SD) or frequency (%). ANOVA and chi-square tests were used where appropriate.

### Distribution of CACS

The distribution of CACS is displayed in Table 2. Overall, a <25th percentile CACS was present in 32.4% of patients. It was observed significantly more often in female patients (38.6% vs. 27.9%, *p* < 0.001). Apart from this difference, the percentiles were evenly distributed between the sexes. Female patients had a CACS of 0 significantly more often than male patients (36.1% vs. 15.6%, *p* < 0.001), whereas CACS values above 100 were significantly more frequent in males.

**Table 2:** Distribution of coronary calcium score.

	Overall (*n* = 1,792)	Male (*n* = 1,030)	Female (*n* = 762)	*p*-value
Calcium score (IQR)	74 (1–413)	148 (11–621)	16 (0–181)	<0.001
CACS percentile (%)				<0.001
<25%	581 (32.4)	287 (27.9)	294 (38.6)	
25%–50%	313 (17.5)	212 (20.6)	101 (13.3)	
50%–75%	367 (20.5)	229 (22.2)	138 (18.1)	
75%–90%	297 (16.6)	168 (16.3)	129 (16.9)	
>90%	234 (13.1)	134 (13.0)	100 (13.1)	
CACS category (%)				<0.001
0	436 (24.3)	161 (15.6)	275 (36.1)	
1–9	170 (9.5)	93 (9.0)	77 (10.1)	
10–99	361 (20.1)	196 (19.0)	165 (21.7)	
100–399	366 (20.4)	238 (23.1)	128 (16.8)	
400–1,000	258 (14.4)	175 (17.0)	83 (10.9)	
>1,000	201 (11.2)	167 (16.2)	34 (4.5)	

Distribution of coronary calcium score (CACS) is stratified by sex. Values are displayed as median (IQR) or frequency (percentage). Wilcoxon and chi-square tests were used where appropriate.

### Test performance of CACS percentile and CACS category

**Figure 2:**
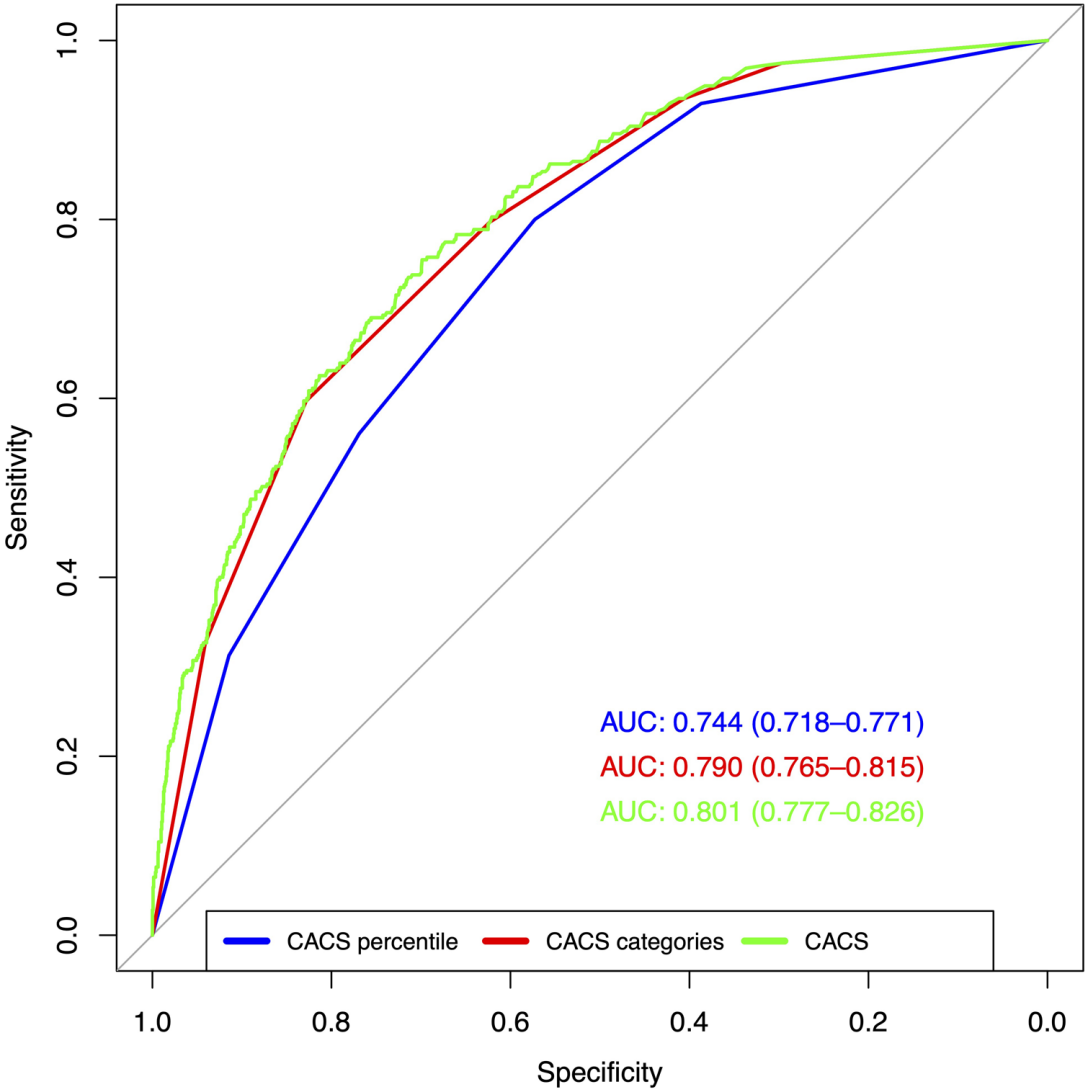
Test performance of CACS percentile, CACS category, and CACS for abnormal PET. ROC indicate the test performance of the three tested variables (CACS percentile, CACS category, and CACS) for abnormal PET (SSS ≥4). The differences between the AUC were statistically significant (*p* < 0.001 each).

The AUC of CACS percentile, CACS category, and CACS for abnormal PET were 0.744 [95% confidence interval (CI): 0.718–0.771], 0.790 (95% CI: 0.765–0.815), and 0.801 (95% CI: 0.777–0.826), respectively. The ROC curves are displayed in Figure 2. The differences between the three curves were statistically significant (*p* < 0.001 each).

**Figure 3:**
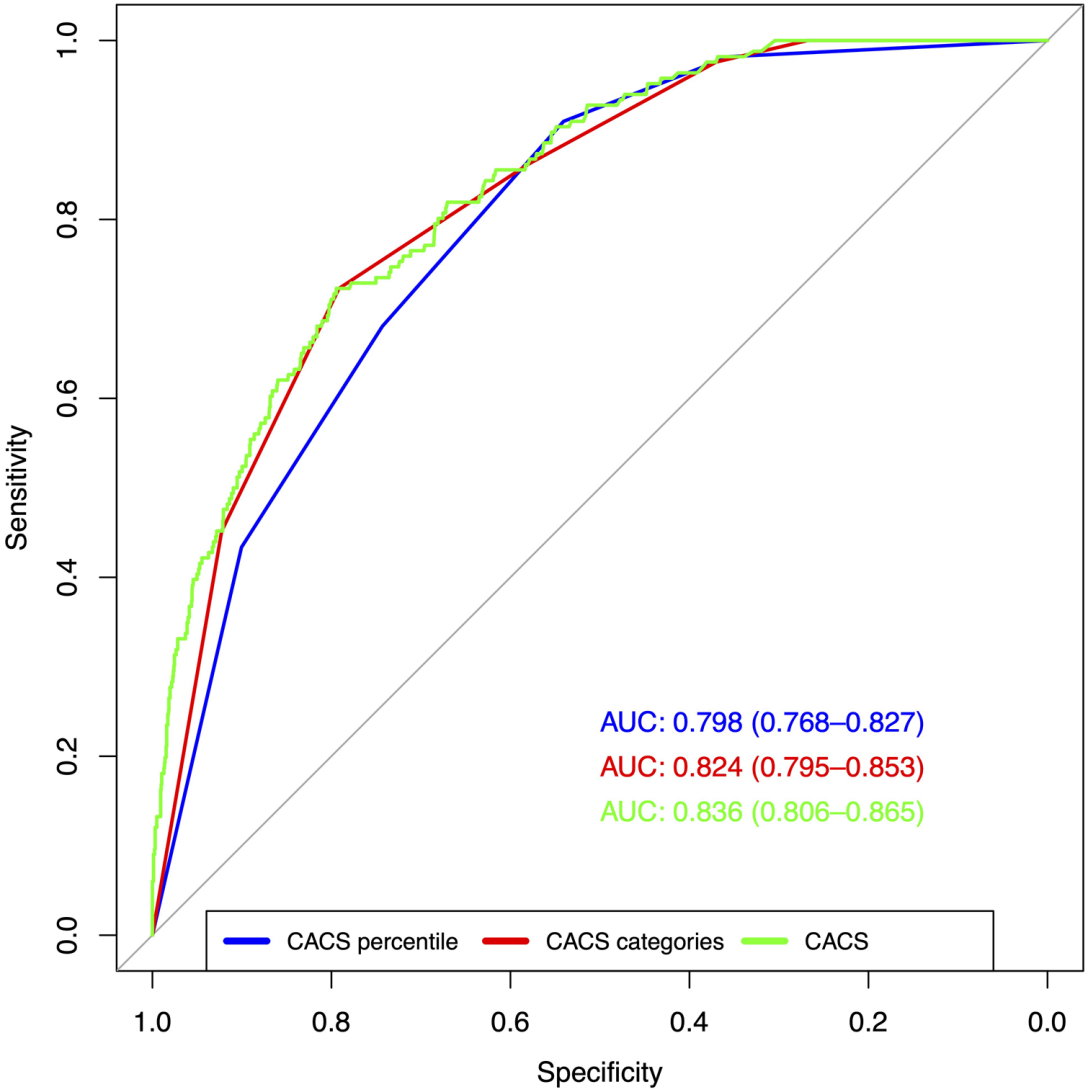
Test performance of CACS percentile, CACS category, and CACS for relevant ischemia (>10%). ROC indicate the test performance of the three tested variables (CACS percentile, CACS category, and CACS) for relevant ischemia (SDS ≥7). CACS had a significantly higher AUC compared to the other variables (*p* < 0.001), but there was no difference between CACS percentile and CACS category (*p* = 0.028).

The AUC of CACS percentile, CACS category, and CACS for relevant ischemia (>10%) were 0.798 (95% CI: 0.768–0.827), 0.824 (95% CI: 0.795–0.853), and 0.836 (95% CI: 0.806–0.865), respectively. The ROC curves are depicted in Figure 3. There was no statistically significant difference between CACS percentile and CACS category (*p* = 0.018), but CACS outperformed both (*p* < 0.001 each).

### Logistic regression analysis

CACS percentile was a strong, independent predictor for abnormal PET in multivariate binary regression analysis. As shown in Table 3, the odds ratio for abnormal PET increased with increased percentile class. Further independent factors (ordered by descending odds ratio) were male sex, LBBB, repolarization abnormalities, typical angina, age, and BMI. The five traditional cardiovascular risk factors were not associated with an abnormal PET result.

**Table 3:** Multivariate analyses of factors associated with abnormal PET.

Variable	Odds ratio	95% confidence interval		*p*-value
BMI	1.032	1.006	1.059	0.015
Age	1.054	1.039	1.07	<0.001
Male sex	3.867	2.828	5.286	<0.001
Symptoms				
Asymptomatic	Ref.	Ref.	Ref.	<0.001
Non-anginal chest pain	0.608	0.35	1.056	0.077
Atypical angina	0.891	0.602	1.32	0.566
Typical angina	1.766	1.203	2.591	0.004
Dyspnea	0.969	0.604	1.554	0.896
Risk factors				
Hypertension	0.864	0.616	1.212	0.397
Hypercholesterolemia	0.825	0.593	1.147	0.252
Diabetes	1.174	0.851	1.619	0.327
Smoking	1.033	0.779	1.369	0.822
Family history	1.088	0.7	1.691	0.708
ECG abnormalities				
LBBB	3.249	1.835	5.752	<0.001
Q wave	1.792	0.987	3.254	0.055
Repolarization abnormalities	1.911	1.305	2.798	<0.001
CACS percentile				
<25	Ref.	Ref.	Ref.	<0.001
25–50	2.806	1.648	4.775	<0.001
50–75	6.001	3.667	9.821	<0.001
75–90	8.878	5.39	14.624	<0.001
>90	23.105	13.751	38.82	<0.001

The table indicates the multivariate binary logistic regression model to predict abnormal PET (SSS ≥ 4).

As shown in Supplementary Table S1, the findings were comparable for relevant ischemia (>10%). Typical angina (OR 3.4) and repolarization abnormalities on a resting ECG were the only significant variables from symptoms and ECG, respectively.

### Distribution of abnormal scan results stratified by CACS percentile

The distribution of abnormal PET and relevant ischemia (>10%) according to the CACS percentile is shown in Panel C of the Graphical Abstract. With a higher percentile, the prevalence of abnormal PET increased significantly from 4.3% (<25th percentile) to 47.4% (>90th percentile) (*p* < 0.001). Similarly, the prevalence of relevant ischemia (>10%) increased significantly from 0.5% (<25th percentile) to 30.8% (>90th percentile) (*p* < 0.001).

### Distribution of abnormal scan results stratified by CACS category

The distribution of abnormal PET and relevant ischemia (>10%) according to CACS category is shown in Panel D of the Graphical Abstract. With a higher CACS category, the prevalence of abnormal PET increased significantly from 2.1% (CACS 0) to 57.7% (CACS >1,000) (*p* < 0.001). Similarly, the prevalence of relevant ischemia (>10%) increased significantly from 0.0% (CACS 0) to 37.3% (CACS > 1,000) (*p* < 0.001).

### Test characteristics to exclude abnormal PET and relevant ischemia (>10%)

Test characteristics are shown in Table 4. The sensitivity of the <25th and 50th percentiles to correctly diagnose abnormal PET was 93.0% and 80.0%, respectively. The sensitivity of CACS categories 1–9 and 1–99 was 96.0% and 81.8%, respectively. The negative predictive values for these cut-offs ranged from 88.1% to 95.7% and the NLR was moderate for all of them (0.182–0.393). The highest sensitivity, NPV, and NLR were observed with CACS 0 (97.5%, 97.9%, and 0.085).

**Table 4:** Test characteristics for abnormal PET or relevant ischemia (>10%).

		Sensitivity	Specificity	NPV	PPV	LR+	LR−	DOR
Abnormal PET (SSS ≥ 4)	<25th percentile	0.930	0.387	0.957	0.273	1.516	0.182	8.331
	<50th percentile	0.800	0.573	0.921	0.316	1.872	0.349	5.362
	CACS 0	0.975	0.297	0.979	0.255	1.387	0.085	16.250
	CACS 1–9	0.960	0.154	0.918	0.280	1.135	0.262	4.332
	CACS 1–99	0.818	0.463	0.881	0.343	1.524	0.393	3.879
Relevant ischemia (>10%)	<25th percentile	0.982	0.355	0.995	0.135	1.523	0.051	29.960
	<50th percentile	0.910	0.541	0.983	0.168	1.980	0.167	11.840
	CACS 0	1.000	0.268	1.000	0.122	1.366	NA	NA
	CACS 1–9	0.976	0.139	0.976	0.137	1.134	0.173	6.565
	CACS 1–99	0.861	0.427	0.957	0.173	1.503	0.325	4.631

NPV, negative predictive value; PPV, positive predictive value; LR+, positive likelihood ratio; LR-, negative likelihood ratio; DOR, diagnostic odds ratio.

Table indicates the test characteristics of different cut-offs (<25th percentile, <50th percentile, CACS 0, CACS 1–9, CACS 1–99) to diagnose/exclude abnormal PET (SSS ≥ 4) and relevant ischemia (SDS ≥ 7). A table including the 95% confidence intervals is displayed in the Supplementary Material (Supplementary Table S2).

The sensitivity of the <25th and <50th percentiles to diagnose relevant ischemia (>10%) was 98.2% and 91.0%, respectively. The sensitivity of the CACS categories 1–9 and 1–99 was 97.6% and 86.1%, respectively. The negative predictive value ranged between 95.7% and 99.5%. The NLR was better if an SDS ≥7 was used as the endpoint (0.051–0.325). Only for <25th percentile was the NLR below 0.1, which is considered good for a rule-out test (23). CACS 0 had 100% sensitivity and NPV.

### Test characteristics in different age groups

As shown in Table 5, the sensitivity of the <25th percentile for abnormal PET ranged between 87.5% and 95.6%. Except for patients aged <50 years, sensitivity and NPV were above 90.9% and 92.4%, respectively.

**Table 5:** Test characteristics for abnormal PET or relevant ischemia (>10%) in different age groups.

Endpoint	Cut-off	Age category	Sensitivity	Specificity	NPV	PPV	LR+	LR−	DOR	*n* =
Abnormal PET (SSS ≥4)	Percentile <25%	<50	0.875	0.701	0.989	0.156	2.924	0.178	16.395	135
		50–59	0.909	0.460	0.969	0.215	1.684	0.198	8.525	394
		60–69	0.956	0.313	0.965	0.263	1.392	0.140	9.935	558
		70–79	0.910	0.323	0.924	0.283	1.344	0.279	4.812	537
		>80	0.946	0.339	0.927	0.417	1.432	0.158	9.072	168
	CACS 0	<50	0.875	0.693	0.989	0.152	2.849	0.180	15.795	135
		50–59	0.945	0.431	0.980	0.212	1.661	0.127	13.112	394
		60–69	0.982	0.243	0.982	0.250	1.298	0.072	18.000	558
		70–79	0.975	0.161	0.957	0.255	1.163	0.152	7.637	537
		>80	1.000	0.161	1.000	0.373	1.191	NA	NA	168
	CACS 1–9	<50	0.875	0.094	0.923	0.057	0.966	1.323	0.730	135
		50–59	0.909	0.153	0.912	0.148	1.074	0.593	1.812	394
		60–69	0.956	0.083	0.881	0.211	1.043	0.526	1.982	558
		70–79	0.975	0.108	0.938	0.243	1.094	0.227	4.824	537
		>80	1.000	0.089	1.000	0.354	1.098			168
Relevant ischemia (>10%)	Percentile <25%	<50	1.000	0.698	1.000	0.133	3.308	NA	NA	135
		50–59	0.964	0.437	0.994	0.116	1.713	0.082	20.971	394
		60–69	1.000	0.282	1.000	0.116	1.393	NA	NA	558
		70–79	0.967	0.300	0.986	0.151	1.383	0.109	12.668	537
		>80	1.000	0.283	1.000	0.181	1.394	NA	NA	168
	CACS 0	<50	1.000	0.690	1.000	0.130	3.225	NA	NA	135
		50–59	1.000	0.407	1.000	0.114	1.687	NA	NA	394
		60–69	1.000	0.216	1.000	0.107	1.275	NA	NA	558
		70–79	1.000	0.147	1.000	0.131	1.172	NA	NA	537
		>80	1.000	0.124	1.000	0.153	1.142	NA	NA	168
	CACS 1–9	<50	0.833	0.093	0.923	0.041	0.919	1.792	0.513	135
		50–59	0.929	0.150	0.965	0.077	1.093	0.475	2.299	394
		60–69	0.979	0.080	0.976	0.091	1.065	0.259	4.109	558
		70–79	1.000	0.101	1.000	0.125	1.112	NA	NA	537
		>80	1.000	0.069	1.000	0.146	1.074	NA	NA	168

NPV, negative predictive value; PPV, positive predictive value; LR+, positive likelihood ratio; LR−, negative likelihood ratio; DOR, diagnostic odds ratio.

Table indicates the test characteristics of different cut-offs (<25th percentile, CACS 0, CACS 1–9) to diagnose/exclude abnormal PET (SSS ≥4) and relevant ischemia (SDS ≥7) in different age groups. A table including the 95% confidence intervals is displayed in the Supplementary Material (Supplementary Table S3). The last column indicates the number of patients in the corresponding age group.

For abnormal PET, CACS 0 had the highest sensitivity and NPV in each age group compared to the <25th percentile and CACS 1–9. Both test characteristic values increased with higher age for CACS 0 and CACS 1–9, but not for the <25th percentile. The sensitivity for abnormal PET in patients <50 years was moderate for all three cut-offs.

For relevant ischemia (>10%), the sensitivity of the <25th percentile ranged between 96.4% and 100% with an NPV between 98.6% and 100%. The sensitivity and NPV of CACS 1–9 improved with increasing age and was ≥97.9%/97.6% in patients 60 years or older. The sensitivity and NPV were excellent (100% each) for CACS 0 in all age groups. This is because no patient with CACS 0 exhibited relevant ischemia (>10%) in the studied patient cohort.

As shown in Supplementary Table S3, the 95% confidence intervals were considerably wide in the lowest age group due to the low prevalence of pathologic findings [*n* = 8 for abnormal PET and *n* = 6 for relevant ischemia (>10%)].

### Test characteristics depending on sex

AUC was higher in females for all cut-offs and endpoints [abnormal PET/CACS percentile: 0.799 vs. 0.728; abnormal PET/CACS category: 0.820 vs. 0.751; relevant ischemia (>10%)/CACS percentile: 0.865 vs. 0.780; relevant ischemia (>10%)/CACS category: 0.880 vs. 0.777].

Test characteristics depending on sex are displayed in Table 6. Overall, the <25th percentile had higher sensitivity and NPV values for abnormal PET and relevant ischemia (>10%) in female compared to male patients. Sensitivity was similar for CACS 0 and CACS 1–9 between male and female patients, but NPV was higher in females.

**Table 6:** Test characteristics for abnormal PET or relevant ischemia (>10%) depending on sex.

Endpoint	Cut-off	Sex	Sensitivity	Specificity	NPV	PPV	LR+	LR−	DOR	*n* =
Abnormal PET (SSS ≥4)	Percentile <25th	Male	0.918	0.348	0.923	0.332	1.409	0.235	5.998	1,030
		Female	0.965	0.430	0.990	0.177	1.695	0.081	20.912	762
	CACS 0	Male	0.978	0.204	0.963	0.303	1.228	0.110	11.211	1,030
		Female	0.965	0.402	0.989	0.170	1.615	0.087	18.627	762
	CACS 1–9	Male	0.959	0.108	0.882	0.275	1.075	0.379	2.833	1,030
		Female	0.965	0.109	0.961	0.121	1.084	0.319	3.401	762
Relevant ischemia (>10%)	Percentile <25th	Male	0.977	0.316	0.990	0.171	1.427	0.073	19.517	1,030
		Female	1.000	0.405	1.000	0.077	1.681	NA	NA	762
	CACS 0	Male	1.000	0.179	1.000	0.150	1.218	NA	NA	1,030
		Female	1.000	0.379	1.000	0.074	1.610	NA	NA	762
	CACS 1–9	Male	0.977	0.100	0.968	0.136	1.085	0.231	4.704	1,030
		Female	0.972	0.105	0.987	0.051	1.086	0.265	4.092	762

NPV, negative predictive value; PPV, positive predictive value; LR+, positive likelihood ratio; LR−, negative likelihood ratio; DOR, diagnostic odds ratio.

Table indicates the test characteristics of different cut-offs (<25th percentile, CACS 0, CACS 1–9) to diagnose/exclude abnormal PET (SSS ≥4) and relevant ischemia (SDS ≥7) stratified by sex. A table including the 95% confidence intervals is displayed in the Supplementary Material (Supplementary Table S4). The last column indicates the number of patients in the corresponding group.

### Safety analysis

The safety analysis using small ischemia (SDS ≥2) revealed similar results compared to abnormal PET (SSS ≥4). The results are displayed in Supplementary Tables S5 and S6 and Supplementary Figure S1.

## Discussion

The main findings of this study are the following: (1) In patients with suspected stable CAD, the 25th percentile, CACS 0, and CACS 1–9 had overall good sensitivity and NPV to rule out abnormal PET and relevant ischemia (>10%). (2) The 25th percentile performed well in all subgroups to exclude relevant ischemia (>10%). (3) To exclude abnormal PET, the test characteristics of the 25th percentile, CACS 0, and CACS 1–9 were sufficient in patients older than 50 years. (4) The 25th percentile had similar test characteristics across all age groups whereas the test characteristics of the absolute CACS categories 0 and 1–9 improved with increasing age. (5) These two cut-offs (25th percentile and CACS category 1–9) could be used in addition to ZCS to help triage 8%–10% more patients with suspected stable CAD into a subgroup without the need for further advanced CAD testing.

In the current study population with suspected stable CAD, an unremarkable scan was present in 80%. Thus, patient preselection was suboptimal and strategies to improve this should be developed and evaluated. In the logistic regression analysis, typical angina, male sex, age, and BMI were the only statistically significant clinical predictors for an abnormal PET. In particular, the traditional five risk factors were not useful in predicting an abnormal finding. In the ECG, LBBB and repolarization abnormalities were associated with abnormal scans. The presence of Q waves tended to be predictive of an abnormal PET (OR 1.8, *p* = 0.051). In contrast, the CACS was a very strong and independent predictor for abnormal PET. As previously shown (17, 20, 24), the prevalence of abnormal scans and ischemia increased with a higher CACS percentile. The abovementioned findings were similar for relevant ischemia (>10%).

The test characteristics of the 25th percentile and CACS 1–9 were good for ruling out abnormal PET and excellent for relevant ischemia (>10%). As expected, ZCS performed better for both endpoints. Still, the sensitivity [in particular for relevant ischemia (>10%)] appears sufficient for clinical use, especially if compared to other non-invasive, (also not perfect) ischemia tests (SPECT 83%–90%, PET 78%–96%, stress CMR 83%–94%, stress echo 80%–89%) (25).

The test characteristics of the 50th percentile and CACS 1–99 were insufficient to diagnose and rule-out abnormal PET. Similarly, the test characteristics for relevant ischemia (>10%) were moderate only. Consequently, no subgroup analyses for these two cut-offs were performed and described.

### Performance in different subgroups

To rule out abnormal PET in patients >50 years, sensitivity and NPV of the <25th percentile were good. The <25th percentile seemed to perform better in female patients compared to male patients, whereas CACS 0 and CACS 1–9 appeared to be unaffected by sex.

The test characteristics for relevant ischemia (>10%) of the 25th percentile and CACS 0 were excellent across all age groups. With increasing age, CACS increases, and the proportion of patients with no/minimal CAC decreases (22). Hence, it is consistent that the sensitivity and NPV of CACS 1–9 improved with increasing age. This trend was identical for relevant ischemia (>10%).

For abnormal PET, all cut-offs had an insufficient sensitivity in patients <50 years (87.5% each). Moreover, the sensitivity of CACS 1–9 was poor in patients <50 years for both diagnostic endpoints [87.5% for abnormal PET, 83.3% for relevant ischemia (>10%)].

This might in part be explained by the low prevalence of abnormal scan results in this rather small age group (leading to wide 95% confidence intervals). However, CACS percentiles and CACS 1–9 appear to be unsuitable in these young patients to be used as gatekeepers due to the relatively higher prevalence of not-yet-calcified plaques.

### Limitation

Certain subgroups (e.g., patients <50 and >80 years) were small. This led to wide 95% confidence intervals in these groups (as shown in Supplementary Tables S3–S4). Therefore, estimates for sensitivity and NPV might be inaccurate, especially in patients below 50 years of age. Hence, the cut-offs should be used with caution in younger patients (<50 years).

The AUC of CACS percentiles and CACS classes were lower compared to the absolute CACS values and published data (7, 24). This is explained by the five available levels that impede a smooth AUC curve compared to a continuous variable. Nevertheless, cut-offs are helpful and frequently used in clinical routines.

Data used for this project arise from a single center without an imaging core laboratory. However, the images were analyzed according to current guidelines by an experienced team of cardiologists and nuclear medicine specialists who reached a consensus. Hence, data interpretation was performed in a standardized and homogeneous way.

SSS ≥4 is a composite endpoint consisting of myocardial scarring and ischemia and thus does not solely describe ischemia. Nevertheless, we find it a useful cut-off to diagnose CAD because it also incorporates scarring from a previous, unrecognized myocardial infarction. Furthermore, it is often used in the literature. The safety analysis using small ischemia (SDS ≥2) revealed similar results.

CACS was compared against PET and not invasive angiography which is regarded as the “gold standard” for the diagnosis of CAD. Thus, there is a residual risk of inaccuracy in the endpoint results (false negative and positive results), but the same applies to invasive angiography, in which only a minority of significant luminal stenoses actually provoke myocardial ischemia (26).

### Comparison with other imaging modalities

Different non-invasive modalities for diagnosis and risk stratification of CAD are available. Computed tomography coronary angiography (CTCA) is an optimal modality for the anatomical assessment of coronary arteries and plaque morphology in patients with low to intermediate pre-test probability. It is the only non-invasive method to detect subclinical atherosclerotic plaques, assess plaque morphology, and identify high-risk features. The high NPV of CTCA is useful for the safe exclusion of CAD which is associated with a long “warranty period” of >10 years (27).

However, its use is limited in patients with significant coronary artery calcification due to blooming artifacts and thus an overestimation of luminal narrowing. In this circumstance, functional ischemia tests as described below are useful. Cardiac magnetic resonance is a well-studied functional ischemia test which is considered the gold standard for myocardial tissue characterization and viability assessment. However, anatomical assessment of coronary arteries is limited due to technical difficulties in delineating the highly mobile and thin coronary arteries. As applicable to all functional ischemia tests, only flow-limiting stenoses can be detected and subclinical CAD remains undetected. Adding CACS to the nuclear myocardial perfusion imaging modalities (SPECT and PET) offers an attractive combination of anatomical and functional CAD assessment. Both components have an excellent performance record for prognostication and complement each other perfectly. Since the amount of coronary calcification correlates with the amount of myocardial ischemia, a severely elevated CACS is helpful to identify patients with a balanced ischemia (24). Furthermore, CACS could be used as a gatekeeper as discussed in this paper.

However, in comparison to CTCA, CACS cannot detect non-calcified plaques which are described in contemporary cohorts to be present in 6%–16% of patients (28–30). Although these plaques are rarely hemodynamically relevant (29), this is a significant drawback of CACS and functional imaging tests since low-attenuation non-calcified plaques are independently associated with myocardial infarctions (31). As demonstrated in the SCOT-HEART trial, detection of subclinical CAD led to more frequent initiation of preventive pharmacological therapies and thus to a reduction of death from coronary heart disease and non-fatal myocardial infarctions (32). In direct comparison to invasive angiography, CTCA exhibited a similar rate of cardiovascular events, but could significantly reduce procedure-related complications (33). In serial CTCA assessment, it was shown that CACS and calcified plaque mass increased irrespective of lipid-lowering therapy, but the progression of non-calcified plaques was halted if LDL cholesterol was significantly reduced (34).

## Conclusion

The 25th percentile and CACS 1–9 reliably excluded abnormal PET and relevant ischemia (>10%) in patients older than 50 years with suspected CAD. Hence, they could extend the scope of application of the well-studied ZCS (or “power of zero”) by 8%–10% to approximately one-third (32%–34%) of referred patients who could be deferred from further testing. Despite slightly lower sensitivity and NPV compared to CACS 0, these cut-offs are comparable with other non-invasive functional ischemia tests. Further studies combining these findings with easily available clinical variables are required to prove the safety and feasibility of the CACS as a gatekeeper prior to advanced cardiac testing.

## Data Availability

The datasets presented in this article are not readily available because of ethical restrictions. Requests to access the datasets should be directed to Michael Zellweger (michael.zellweger@usb.ch).
