# Supplementary material for: Diagnostic utility of coronary artery calcium score percentiles and categories to exclude abnormal scans and relevant ischemia in rubidium positron emission tomography
Source: Front Cardiovasc Med. 2024 Sep 23;11:1467916. doi: 10.3389/fcvm.2024.1467916 (PMC11460730; doi:10.3389/fcvm.2024.1467916)
Supplement: Supplementary file 1 [file Datasheet1.pdf]

Supplementary materials

PET image reconstruction details:

For reconstruction, an ordered subset expectation maximization algorithm (OSEM, matrix 512 x 512, 3 iterations, 21 subsets, gauss-filtering, 8 mm full-width half-maximum) was used for static and dynamic images. Random, scatter, attenuation and decay corrections were automatically applied to the emission data. Automatic motion correction was enabled. The following framesets were used for reconstruction of dynamic images: 1s delay, 12x10 s, 4x30 s, 1x60 s, 1x120s. The last four minutes of acquisition were used for reconstruction of all images (static and ECG-gated).

Search term used on Pubmed:

(((coronary calcium score) OR (CACS) OR (coronary calcium)) AND (ischemia)) AND ((percentile) OR (quartile))

Supplementary Table S1: Factors associated with relevant ischemia

Variable	Odds ratio	95% confidence interval		p-value
BMI	1.024	0.988	1.061	0.192
Age	1.046	1.026	1.066	<0.001
Male sex	4.241	2.725	6.601	<0.001
**Symptoms**				
Asymptomatic	Ref.	Ref.	Ref.	<0.001
Non-anginal chest pain	1.1	0.528	2.292	0.798
Atypical angina	1.156	0.664	2.015	0.608
Typical angina	3.368	2.014	5.634	<0.001
Dyspnea	0.864	0.444	1.679	0.665
**Risk factors**				
Hypertension	0.788	0.495	1.254	0.314
Hypercholesterolemia	1.004	0.641	1.57	0.988
Diabetes	1.357	0.889	2.072	0.156
Smoking	1.109	0.754	1.631	0.601
Family history	0.835	0.453	1.538	0.563
**ECG abnormalities**				
LBBB	1.568	0.669	3.674	0.301
Q wave	1.829	0.885	3.782	0.103
Repolarization abnormalities	2.34	1.446	3.784	<0.001
**CACS Percentile**				
< 25.	Ref.	Ref.	Ref.	<0.001
25. - 50.	5.263	1.453	19.067	0.011
50. - 75.	18.492	5.599	61.081	<0.001
75. - 90.	27.066	8.194	89.403	<0.001
>90.	90.74	27.608	298.234	<0.001

The table indicates the binary logistic regression model to predict relevant ischemia (SDS $\geq$7).

Supplementary Table S2: Test characteristics to diagnose and exclude abnormal PET or relevant ischemia

		Sensitivity	Specificity	NPV	PPV	LR+	LR-	DOR
SSS ≥ 4	<25th percentile	0.930 (0.898 - 0.952)	0.387 (0.362 - 0.412)	0.957 (0.937 - 0.971)	0.273 (0.248 - 0.298)	1.516 (1.442 - 1.594)	0.182 (0.124 - 0.267)	8.331 (5.472 - 12.681)
	<50th percentile	0.800 (0.755 - 0.838)	0.573 (0.547 - 0.598)	0.921 (0.901 - 0.937)	0.316 (0.287 - 0.347)	1.872 (1.730 - 2.027)	0.349 (0.282 - 0.432)	5.362 (4.051 - 7.096)
	CACS 0	0.975 (0.953 - 0.987)	0.297 (0.274 - 0.321)	0.979 (0.961 - 0.989)	0.255 (0.233 - 0.279)	1.387 (1.336 - 1.440)	0.085 (0.045 - 0.163)	16.253 (8.306 - 31.806)
	CACS 1-9	0.960 (0.933 - 0.976)	0.154 (0.133 - 0.178)	0.918 (0.867 - 0.95)	0.280 (0.255 - 0.306)	1.135 (1.097 - 1.174)	0.262 (0.154 - 0.446)	4.332 (2.471 - 7.594)
	CACS 1-99	0.818 (0.774 - 0.855)	0.463 (0.433 - 0.494)	0.881 (0.851 - 0.906)	0.343 (0.311 - 0.376)	1.524 (1.413 - 1.644)	0.393 (0.311 - 0.496)	3.879 (2.874 - 5.234)
SDS ≥ 7	<25th percentile	0.982 (0.948 - 0.994)	0.355 (0.333 - 0.379)	0.995 (0.985 - 0.998)	0.135 (0.117 - 0.155)	1.523 (1.461 - 1.588)	0.051 (0.017 - 0.156)	29.966 (9.522 - 94.305)
	<50th percentile	0.910 (0.856 - 0.944)	0.541 (0.516 - 0.565)	0.983 (0.973 - 0.99)	0.168 (0.145 - 0.194)	1.980 (1.844 - 2.126)	0.167 (0.103 - 0.271)	11.846 (6.906 - 20.317)
	CACS 0	1.000 (0.977 - 1.000)	0.268 (0.247 - 0.290)	1.000 (0.991 - 1.000)	0.122 (0.106 - 0.141)	1.366 (1.327 - 1.407)	NA	NA
	CACS 1-9	0.976 (0.940 - 0.991)	0.139 (0.121 - 0.160)	0.976 (0.941 - 0.991)	0.137 (0.118 - 0.157)	1.134 (1.097 - 1.172)	0.173 (0.065 - 0.459)	6.565 (2.402 - 17.945)
	CACS 1-99	0.861 (0.801 - 0.906)	0.427 (0.399 - 0.455)	0.957 (0.936 - 0.971)	0.173 (0.149 - 0.201)	1.503 (1.39 - 1.625)	0.325 (0.221 - 0.477)	4.631 (2.938 - 7.300)

Table indicates the test characteristics of different cut-offs (<25th percentile, <50th percentile, CACS 0, CACS 1-9, CACS 1-99) to diagnose/exclude abnormal PET (SSS ≥ 4) and relevant ischemia (SDS ≥ 7). The values in brackets correspond to the 95% confidence interval. NPV: negative predictive value. PPV: positive predictive value. LR+: positive likelihood ratio. LR-: negative likelihood ratio. DOR: diagnostic odds ratio.

Supplementary Table S3: Test characteristics to diagnose and exclude abnormal PET or relevant ischemia in different age groups

Endpoint	Cut-off	Age category	Sensitivity	Specificity	NPV	PPV	LR+	LR-	DOR
**Abnormal PET (SSS ≥4)**	Percentile <25%	**<50**	0.875 (0.529 - 0.978)	0.701 (0.616 - 0.774)	0.989 (0.940 - 0.998)	0.156 (0.077 - 0.288)	2.924 (2.013 - 4.248)	0.178 (0.028 - 1.120)	16.395 (1.949 - 137.882)
		**50-59**	0.909 (0.804 - 0.961)	0.460 (0.408 - 0.513)	0.969 (0.929 - 0.987)	0.215 (0.167 - 0.272)	1.684 (1.480 - 1.916)	0.198 (0.085 - 0.459)	8.525 (3.317 - 21.906)
		**60-69**	0.956 (0.901 - 0.981)	0.313 (0.272 - 0.358)	0.965 (0.921 - 0.985)	0.263 (0.223 - 0.308)	1.392 (1.292 - 1.499)	0.140 (0.059 - 0.334)	9.935 (3.965 - 24.894)
		**70-79**	0.910 (0.846 - 0.949)	0.323 (0.280 - 0.369)	0.924 (0.869 - 0.957)	0.283 (0.241 - 0.33)	1.344 (1.232 - 1.466)	0.279 (0.156 - 0.499)	4.812 (2.505 - 9.244)
		**>80**	0.946 (0.854 - 0.982)	0.339 (0.258 - 0.431)	0.927 (0.806 - 0.975)	0.417 (0.335 - 0.504)	1.432 (1.237 - 1.659)	0.158 (0.051 - 0.489)	9.072 (2.659 - 30.950)
	CACS 0	**<50**	0.875 (0.529 - 0.978)	0.693 (0.608 - 0.766)	0.989 (0.939 - 0.998)	0.152 (0.076 - 0.282)	2.849 (1.968 - 4.125)	0.180 (0.029 - 1.133)	15.795 (1.879 - 132.771)
		**50-59**	0.945 (0.851 - 0.981)	0.431 (0.379 - 0.484)	0.980 (0.942 - 0.993)	0.212 (0.166 - 0.268)	1.661 (1.484 - 1.858)	0.127 (0.042 - 0.383)	13.112 (4.015 - 42.820)
		**60-69**	0.982 (0.938 - 0.995)	0.243 (0.206 - 0.285)	0.982 (0.936 - 0.995)	0.250 (0.212 - 0.292)	1.298 (1.225 - 1.376)	0.072 (0.018 - 0.288)	18.000 (4.373 - 74.092)
		**70-79**	0.975 (0.930 - 0.992)	0.161 (0.129 - 0.200)	0.957 (0.881 - 0.985)	0.255 (0.217 - 0.296)	1.163 (1.106 - 1.224)	0.152 (0.049 - 0.476)	7.637 (2.358 - 24.735)
		**>80**	1.000 (0.936 - 1.000)	0.161 (0.104 - 0.240)	1.000 (0.824 - 1.000)	0.373 (0.300 - 0.453)	1.191 (1.099 - 1.292)	NA	NA
	CACS 1-9	**<50**	0.875 (0.529 - 0.978)	0.094 (0.055 - 0.158)	0.923 (0.667 - 0.986)	0.057 (0.028 - 0.114)	0.966 (0.739 - 1.263)	1.323 (0.196 - 8.941)	0.730 (0.083 - 6.449)
		**50-59**	0.909 (0.804 - 0.961)	0.153 (0.119 - 0.196)	0.912 (0.811 - 0.962)	0.148 (0.114 - 0.19)	1.074 (0.976 - 1.181)	0.593 (0.248 - 1.418)	1.812 (0.690 - 4.759)
		**60-69**	0.956 (0.901 - 0.981)	0.083 (0.061 - 0.113)	0.881 (0.750 - 0.948)	0.211 (0.178 - 0.249)	1.043 (0.994 - 1.095)	0.526 (0.212 - 1.309)	1.982 (0.761 - 5.163)
		**70-79**	0.975 (0.930 - 0.992)	0.108 (0.082 - 0.142)	0.938 (0.832 - 0.979)	0.243 (0.207 - 0.283)	1.094 (1.047 - 1.143)	0.227 (0.072 - 0.717)	4.824 (1.472 - 15.807)
		**>80**	1.000 (0.936 - 1.000)	0.089 (0.049 - 0.157)	1.000 (0.722 - 1.000)	0.354 (0.284 - 0.432)	1.098 (1.036 - 1.164)		
**Relevant ischemia (SDS ≥7)**	Percentile <25%	**<50**	1.000 (0.610 - 1.000)	0.698 (0.614 - 0.77)	1.000 (0.959 - 1.000)	0.133 (0.063 - 0.262)	3.308 (2.545 - 4.299)	NA	NA
		**50-59**	0.964 (0.823 - 0.994)	0.437 (0.387 - 0.488)	0.994 (0.966 - 0.999)	0.116 (0.081 - 0.163)	1.713 (1.527 - 1.922)	0.082 (0.012 - 0.562)	20.971 (2.819 - 155.979)
		**60-69**	1.000 (0.926 - 1.000)	0.282 (0.245 - 0.323)	1.000 (0.974 - 1.000)	0.116 (0.089 - 0.150)	1.393 (1.320 - 1.471)	NA	NA
		**70-79**	0.967 (0.888 - 0.991)	0.300 (0.261 - 0.343)	0.986 (0.951 - 0.996)	0.151 (0.119 - 0.189)	1.383 (1.283 - 1.490)	0.109 (0.028 - 0.429)	12.668 (3.054 - 52.554)
		**>80**	1.000 (0.857 - 1.000)	0.283 (0.216 - 0.361)	1.000 (0.914 - 1.000)	0.181 (0.124 - 0.257)	1.394 (1.259 - 1.544)	NA	NA
	CACS 0	**<50**	1.000 (0.610 - 1.000)	0.690 (0.606 - 0.763)	1.000 (0.959 - 1.000)	0.130 (0.061 - 0.257)	3.225 (2.493 - 4.172)	NA	NA
		**50-59**	1.000 (0.879 - 1.000)	0.407 (0.358 - 0.458)	1.000 (0.975 - 1.000)	0.114 (0.080 - 0.16)	1.687 (1.549 - 1.836)	NA	NA
		**60-69**	1.000 (0.926 - 1.000)	0.216 (0.182 - 0.253)	1.000 (0.966 - 1.000)	0.107 (0.082 - 0.139)	1.275 (1.218 - 1.334)	NA	NA
		**70-79**	1.000 (0.941 - 1.000)	0.147 (0.118 - 0.182)	1.000 (0.948 - 1.000)	0.131 (0.103 - 0.164)	1.172 (1.129 - 1.217)	NA	NA
		**>80**	1.000 (0.857 - 1.000)	0.124 (0.080 - 0.188)	1.000 (0.824 - 1.000)	0.153 (0.104 - 0.22)	1.142 (1.074 - 1.214)	NA	NA
	CACS 1-9	**<50**	0.833 (0.436 - 0.970)	0.093 (0.054 - 0.156)	0.923 (0.667 - 0.986)	0.041 (0.018 - 0.092)	0.919 (0.640 - 1.32)	1.792 (0.277 - 11.608)	0.513 (0.055 - 4.758)
		**50-59**	0.929 (0.774 - 0.980)	0.150 (0.117 - 0.191)	0.965 (0.881 - 0.990)	0.077 (0.053 - 0.111)	1.093 (0.978 - 1.222)	0.475 (0.122 - 1.847)	2.299 (0.530 - 9.964)
		**60-69**	0.979 (0.891 - 0.996)	0.080 (0.060 - 0.107)	0.976 (0.877 - 0.996)	0.091 (0.069 - 0.119)	1.065 (1.014 - 1.118)	0.259 (0.036 - 1.843)	4.109 (0.553 - 30.55)
		**70-79**	1.000 (0.941 - 1.000)	0.101 (0.077 - 0.131)	1.000 (0.926 - 1.000)	0.125 (0.098 - 0.157)	1.112 (1.079 - 1.146)	NA	NA
		**>80**	1.000 (0.857 - 1.000)	0.069 (0.038 - 0.122)	1.000 (0.722 - 1.000)	0.146 (0.099 - 0.209)	1.074 (1.028 - 1.123)	NA	NA

Table indicates the test characteristics of different cut-offs (<25th percentile, CACS 0, CACS 1-9) to diagnose/exclude abnormal PET (SSS ≥4) and relevant ischemia (SDS ≥7). The values in brackets correspond to the 95% confidence interval.

NPV: negative predictive value. PPV: positive predictive value. LR+: positive likelihood ratio. LR-: negative likelihood ratio. DOR: diagnostic odds ratio.

Supplementary Table S4: Test characteristics for abnormal PET or relevant ischemia depending on sex

Endpoint	Cut-off	Sex	Sensitivity	Specificity	NPV	PPV	LR+	LR-	DOR
**Abnormal PET (SSS ≥4)**	Percentile <25th	**Male**	0.918 (0.879 - 0.945)	0.348 (0.315 - 0.383)	0.923 (0.887 - 0.949)	0.332 (0.300 - 0.367)	1.409 (1.323 - 1.500)	0.235 (0.156 - 0.355)	5.998 (3.783 - 9.510)
		**Female**	0.965 (0.902 - 0.988)	0.430 (0.394 - 0.468)	0.990 (0.970 - 0.997)	0.177 (0.145 - 0.215)	1.695 (1.569 - 1.830)	0.081 (0.027 - 0.247)	20.912 (6.543 - 66.832)
	CACS 0	**Male**	0.978 (0.952 - 0.990)	0.204 (0.177 - 0.234)	0.963 (0.921 - 0.983)	0.303 (0.273 - 0.334)	1.228 (1.179 - 1.278)	0.110 (0.049 - 0.245)	11.211 (4.897 - 25.666)
		**Female**	0.965 (0.902 - 0.988)	0.402 (0.366 - 0.440)	0.989 (0.968 - 0.996)	0.170 (0.140 - 0.206)	1.615 (1.500 - 1.739)	0.087 (0.028 - 0.265)	18.627 (5.827 - 59.542)
	CACS 1-9	**Male**	0.959 (0.928 - 0.977)	0.108 (0.088 - 0.132)	0.882 (0.801 - 0.933)	0.275 (0.248 - 0.305)	1.075 (1.038 - 1.113)	0.379 (0.205 - 0.701)	2.833 (1.485 - 5.401)
		**Female**	0.965 (0.902 - 0.988)	0.109 (0.088 - 0.135)	0.961 (0.892 - 0.987)	0.121 (0.099 - 0.148)	1.084 (1.033 - 1.137)	0.319 (0.103 - 0.989)	3.401 (1.048 - 11.033)
**Relevant ischemia (SDS ≥7)**	Percentile <25th	**Male**	0.977 (0.934 - 0.992)	0.316 (0.286 - 0.347)	0.990 (0.970 - 0.996)	0.171 (0.146 - 0.2)	1.427 (1.355 - 1.503)	0.073 (0.024 - 0.225)	19.517 (6.158 - 61.854)
		**Female**	1.000 (0.904 - 1.000)	0.405 (0.370 - 0.441)	1.000 (0.987 - 1.000)	0.077 (0.056 - 0.105)	1.681 (1.583 - 1.784)	NA	NA
	CACS 0	**Male**	1.000 (0.971 - 1.000)	0.179 (0.155 - 0.205)	1.000 (0.977 - 1.000)	0.150 (0.127 - 0.175)	1.218 (1.181 - 1.256)	NA	NA
		**Female**	1.000 (0.904 - 1.000)	0.379 (0.344 - 0.415)	1.000 (0.986 - 1.000)	0.074 (0.054 - 0.101)	1.610 (1.521 - 1.704)	NA	NA
	CACS 1-9	**Male**	0.977 (0.934 - 0.992)	0.100 (0.082 - 0.121)	0.968 (0.909 - 0.989)	0.136 (0.115 - 0.159)	1.085 (1.049 - 1.123)	0.231 (0.074 - 0.718)	4.704 (1.467 - 15.086)
		**Female**	0.972 (0.858 - 0.995)	0.105 (0.084 - 0.129)	0.987 (0.930 - 0.998)	0.051 (0.037 - 0.070)	1.086 (1.022 - 1.154)	0.265 (0.038 - 1.854)	4.092 (0.553 - 30.296)

Table indicates the test characteristics of different cut-offs (<25th percentile, CACS 0, CACS 1-9) to diagnose/exclude abnormal PET (SSS ≥4) and relevant ischemia (SDS ≥7) depending on sex. The values in brackets correspond to the 95% confidence interval.

NPV: negative predictive value. PPV: positive predictive value. LR+: positive likelihood ratio. LR-: negative likelihood ratio. DOR: diagnostic odds ratio.

Supplementary Table S5: Sensitivity analysis of the different cut-offs for small ischemia SDS ≥2

Cut-off		Sensitivity	Specificity	NPV	PPV	LR+	LR-	DOR
**Overall**								
**<25th percentile**		0.929	0.387	0.957	0.272	1.515	0.183	8.296
**<50th percentile**		0.788	0.570	0.916	0.311	1.831	0.372	4.922
**CACS 0**		0.977	0.298	0.982	0.255	1.392	0.076	18.328
**CACS 1-9**		0.960	0.108	0.918	0.210	1.077	0.365	2.955
**CACS 1-99**		0.797	0.622	0.926	0.342	2.110	0.327	6.456
**Age group**								
**Percentile <25%**	<50	0.875	0.701	0.989	0.156	2.924	0.178	16.395
	50-59	0.918	0.455	0.975	0.193	1.685	0.179	9.395
	60-69	0.948	0.312	0.958	0.263	1.377	0.167	8.220
	70-79	0.907	0.326	0.917	0.298	1.346	0.285	4.715
	>80	0.962	0.339	0.951	0.402	1.456	0.111	13.086
**CACS 0**	<50	0.875	0.693	0.989	0.152	2.849	0.180	15.795
	50-59	0.959	0.426	0.987	0.192	1.671	0.096	17.447
	60-69	0.983	0.244	0.982	0.252	1.299	0.071	18.215
	70-79	0.977	0.164	0.957	0.270	1.169	0.142	8.252
	>80	1.000	0.157	1.000	0.353	1.186	NA	NA
**CACS 1-9**	<50	0.875	0.094	0.923	0.057	0.966	1.323	0.730
	50-59	0.918	0.154	0.930	0.134	1.085	0.531	2.042
	60-69	0.948	0.081	0.857	0.211	1.032	0.642	1.607
	70-79	0.977	0.110	0.938	0.258	1.098	0.211	5.207
	>80	1.000	0.087	1.000	0.335	1.095	NA	NA
**Sex**								
**Percentile <25th**	Male	0.925	0.349	0.930	0.331	1.422	0.215	6.608
	Female	0.943	0.429	0.983	0.177	1.651	0.133	12.461
**CACS 0**	Male	0.985	0.205	0.975	0.301	1.240	0.073	16.942
	Female	0.965	0.402	0.989	0.170	1.615	0.087	18.627
**CACS 1-9**	Male	0.959	0.107	0.882	0.272	1.074	0.385	2.787
	Female	0.966	0.110	0.961	0.124	1.085	0.311	3.494

Table indicates the test characteristics of different cut-offs to diagnose/exclude small ischemia (SDS ≥2) in different subgroups. A table including the 95% confidence intervals is displayed in the Supplemental Table S6.

NPV: negative predictive value. PPV: positive predictive value. LR+: positive likelihood ratio. LR-: negative likelihood ratio. DOR: diagnostic odds ratio.

Supplementary Table S6: Sensitivity analysis of the different cut-offs for small ischemia SDS ≥2 with 95%-confidence intervals

		Sensitivity	Specificity	NPV	PPV	LR+	LR-	DOR
**Overall**								
**<25th percentile**		0.929 (0.898 to 0.952)	0.387 (0.362 to 0.412)	0.957 (0.937 to 0.971)	0.272 (0.247 to 0.297)	1.515 (1.441 to 1.000.593)	0.183 (0.124 to 0.268)	8.296 (5.449 to 1.0002.629)
**<50th percentile**		0.788 (0.743 to 0.827)	0.570 (0.544 to 0.595)	0.916 (0.896 to 0.933)	0.311 (0.281 to 0.342)	1.831 (1.69 to 1.000.984)	0.372 (0.303 to 0.457)	4.922 (3.737 to 6.483)
**CACS 0**		0.977 (0.956 to 0.989)	0.298 (0.275 to 0.322)	0.982 (0.964 to 0.991)	0.255 (0.233 to 0.279)	1.392 (1.341 to 1.000.444)	0.076 (0.038 to 0.151)	18.328 (9.011 to 37.277)
**CACS 1-9**		0.960 (0.935 to 0.976)	0.108 (0.093 to 0.126)	0.918 (0.867 to 0.95)	0.210 (0.191 to 0.23)	1.077 (1.048 to 1.000.108)	0.365 (0.214 to 0.622)	2.955 (1.688 to 5.172)
**CACS 1-99**		0.797 (0.752 to 0.835)	0.622 (0.597 to 0.647)	0.926 (0.907 to 0.94)	0.342 (0.31 to 0.375)	2.110 (1.938 to 2.296)	0.327 (0.265 to 0.403)	6.456 (4.88 to 8.541)
**Age group**								
**Percentile <25%**	<50	0.875 (0.529 to 0.978)	0.701 (0.616 to 0.774)	0.989 (0.94 to 0.998)	0.156 (0.077 to 0.288)	2.924 (2.013 to 4.248)	0.178 (0.028 to 1.000.12)	16.395 (1.949 to 1.00037.882)
	50-59	0.918 (0.808 to 0.968)	0.455 (0.403 to 0.508)	0.975 (0.938 to 0.99)	0.193 (0.148 to 0.249)	1.685 (1.483 to 1.000.915)	0.179 (0.07 to 0.462)	9.395 (3.306 to 26.696)
	60-69	0.948 (0.891 to 0.976)	0.312 (0.27 to 0.356)	0.958 (0.912 to 0.981)	0.263 (0.223 to 0.308)	1.377 (1.276 to 1.000.485)	0.167 (0.076 to 0.369)	8.220 (3.527 to 1.0009.157)
	70-79	0.907 (0.844 to 0.946)	0.326 (0.282 to 0.373)	0.917 (0.861 to 0.952)	0.298 (0.255 to 0.346)	1.346 (1.233 to 1.000.468)	0.285 (0.164 to 0.498)	4.715 (2.514 to 8.846)
	>80	0.962 (0.872 to 0.99)	0.339 (0.259 to 0.43)	0.951 (0.839 to 0.987)	0.402 (0.32 to 0.489)	1.456 (1.264 to 1.000.677)	0.111 (0.028 to 0.444)	13.086 (3.025 to 56.606)
**CACS 0**	<50	0.875 (0.529 to 0.978)	0.693 (0.608 to 0.766)	0.989 (0.939 to 0.998)	0.152 (0.076 to 0.282)	2.849 (1.968 to 4.125)	0.180 (0.029 to 1.000.133)	15.795 (1.879 to 1.00032.771)
	50-59	0.959 (0.863 to 0.989)	0.426 (0.375 to 0.479)	0.987 (0.952 to 0.996)	0.192 (0.147 to 0.246)	1.671 (1.501 to 1.000.861)	0.096 (0.025 to 0.374)	17.447 (4.171 to 72.985)
	60-69	0.983 (0.939 to 0.995)	0.244 (0.206 to 0.286)	0.982 (0.936 to 0.995)	0.252 (0.214 to 0.294)	1.299 (1.226 to 1.000.377)	0.071 (0.018 to 0.285)	18.215 (4.426 to 74.969)
	70-79	0.977 (0.934 to 0.992)	0.164 (0.131 to 0.203)	0.957 (0.881 to 0.985)	0.270 (0.232 to 0.312)	1.169 (1.111 to 1.000.229)	0.142 (0.045 to 0.443)	8.252 (2.55 to 26.71)
	>80	1.000 (0.932 to 1.000)	0.157 (0.101 to 0.234)	1.000 (0.824 to 1.000)	0.353 (0.281 to 0.433)	1.186 (1.096 to 1.000.283)	NA	NA
**CACS 1-9**	<50	0.875 (0.529 to 0.978)	0.094 (0.055 to 0.158)	0.923 (0.667 to 0.986)	0.057 (0.028 to 0.114)	0.966 (0.739 to 1.000.263)	1.323 (0.196 to 8.941)	0.730 (0.083 to 6.449)
	50-59	0.918 (0.808 to 0.968)	0.154 (0.119 to 0.195)	0.930 (0.833 to 0.972)	0.134 (0.101 to 0.174)	1.085 (0.987 to 1.000.193)	0.531 (0.201 to 1.000.404)	2.042 (0.705 to 5.915)
	60-69	0.948 (0.891 to 0.976)	0.081 (0.059 to 0.11)	0.857 (0.722 to 0.933)	0.211 (0.178 to 0.249)	1.032 (0.98 to 1.000.086)	0.642 (0.277 to 1.000.487)	1.607 (0.66 to 3.912)
	70-79	0.977 (0.934 to 0.992)	0.110 (0.083 to 0.144)	0.938 (0.832 to 0.979)	0.258 (0.221 to 0.298)	1.098 (1.051 to 1.000.146)	0.211 (0.067 to 0.667)	5.207 (1.59 to 1.0007.049)
	>80	1.000 (0.932 to 1.000)	0.087 (0.048 to 0.153)	1.000 (0.722 to 1.000)	0.335 (0.267 to 0.412)	1.095 (1.035 to 1.000.159)	NA	NA
**Sex**								
**Percentile <25th**	Male	0.925 (0.887 to 0.951)	0.349 (0.316 to 0.384)	0.930 (0.895 to 0.954)	0.331 (0.298 to 0.366)	1.422 (1.336 to 1.000.513)	0.215 (0.14 to 0.332)	6.608 (4.091 to 1.0000.672)
	Female	0.943 (0.874 to 0.975)	0.429 (0.392 to 0.466)	0.983 (0.961 to 0.993)	0.177 (0.145 to 0.215)	1.651 (1.519 to 1.000.794)	0.133 (0.056 to 0.312)	12.461 (4.989 to 31.122)
**CACS 0**	Male	0.985 (0.962 to 0.994)	0.205 (0.178 to 0.236)	0.975 (0.938 to 0.99)	0.301 (0.272 to 0.333)	1.240 (1.192 to 1.000.289)	0.073 (0.027 to 0.195)	16.942 (6.214 to 46.186)
	Female	0.965 (0.902 to 0.988)	0.402 (0.366 to 0.44)	0.989 (0.968 to 0.996)	0.170 (0.14 to 0.206)	1.615 (1.5 to 1.000.739)	0.087 (0.028 to 0.265)	18.627 (5.827 to 59.542)
**CACS 1-9**	Male	0.959 (0.927 to 0.977)	0.107 (0.087 to 0.131)	0.882 (0.801 to 0.933)	0.272 (0.245 to 0.302)	1.074 (1.037 to 1.000.112)	0.385 (0.209 to 0.712)	2.787 (1.462 to 5.316)
	Female	0.966 (0.905 to 0.988)	0.110 (0.088 to 0.136)	0.961 (0.892 to 0.987)	0.124 (0.101 to 0.151)	1.085 (1.035 to 1.000.138)	0.311 (0.1 to 0.964)	3.494 (1.078 to 1.0001.332)

Table indicates the test characteristics of different cut-offs to diagnose/exclude small ischemia (SDS ≥2) in different subgroups. The values in brackets correspond to the lower and upper 95% confidence interval.

NPV: negative predictive value. PPV: positive predictive value. LR+: positive likelihood ratio. LR-: negative likelihood ratio. DOR: diagnostic odds ratio.

Supplementary Figure 1: Test performance of CACS percentile, CACS category and CACS for small ischemia (SDS ≥2)

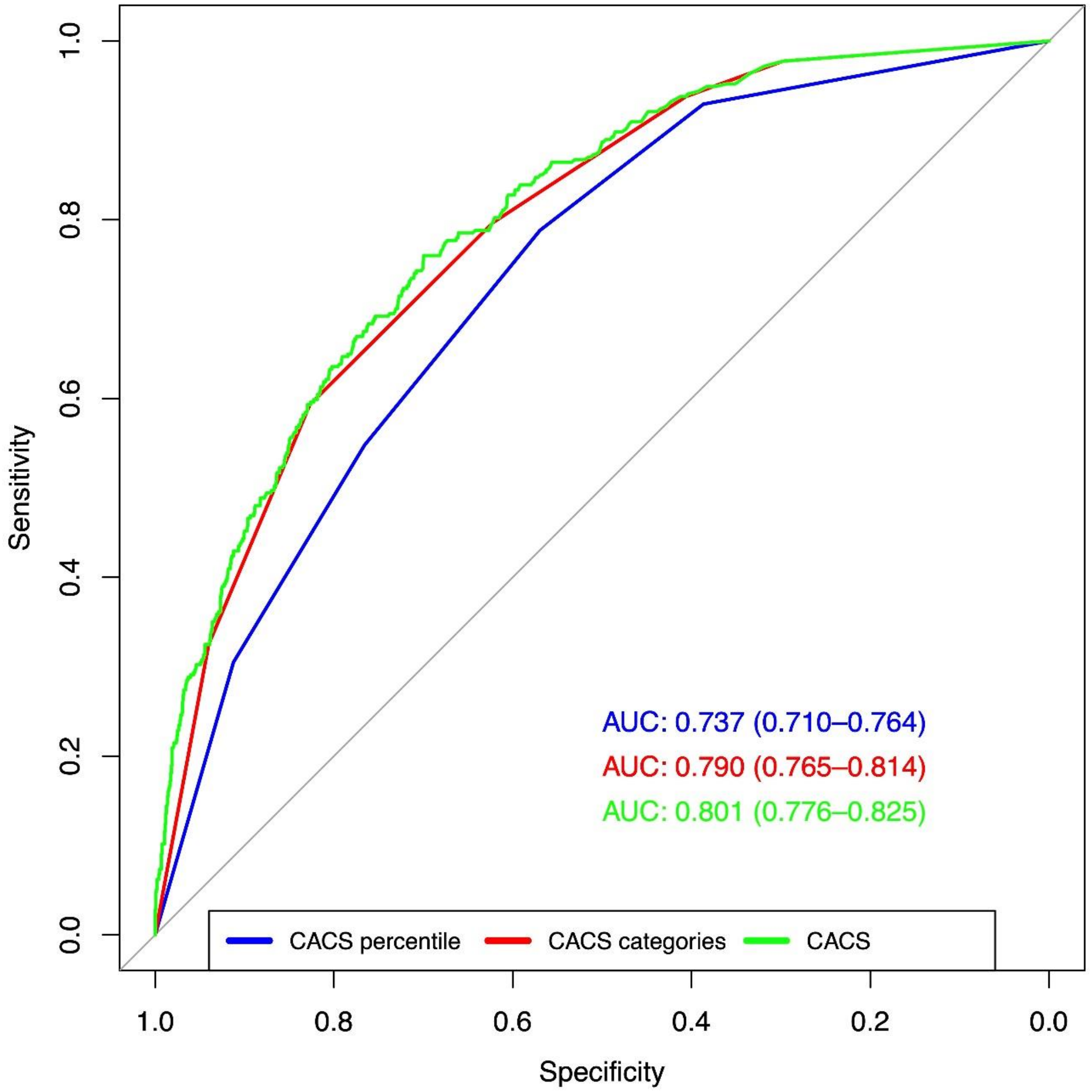

Receiver operator curves (ROC) indicate the test performance of the three tested variables (CACS percentile, CACS category and CACS) for small ischemia (SDS ≥2). CACS had a significantly higher AUC compared to the other variables. All differences were statistically significant ($p<0.001$).
